# Epidemiological, pathological, and molecular characteristics of lung cancer in Casablanca, Morocco: a retrospective study

**DOI:** 10.11604/pamj.2025.51.79.44198

**Published:** 2025-07-25

**Authors:** Zineb Khadrouf, Khadija Khadiri, Abderrahmane Mellouki, Amina Essalihi, Oumaima Bouchra, Majda Taoudi Benchekroun, Abdallah Naya, Mehdi Karkouri

**Affiliations:** 1 Laboratory of Immunology and Biodiversity, Faculty of Sciences Ain Chock, Hassan II University, Casablanca, Morocco,; 2 Department of Pathology, Ibn Rochd University Hospital, Casablanca, Morocco,; 3 Laboratory of Cellular and Molecular Pathology, Faculty of Medicine and Pharmacy, Hassan II University, Casablanca, Morocco.

**Keywords:** Lung cancer, epidemiology, lung adenocarcinoma, Morocco

## Abstract

**Introduction:**

lung cancer remains a significant global health concern, responsible for the highest number of cancer-related deaths worldwide. In Morocco, it is the most prevalent cancer among men. This study aimed to describe the epidemiological characteristics of lung cancer and to explore the molecular profile of lung adenocarcinoma cases.

**Methods:**

a retrospective analysis was carried out over a 24-month period, from January 2020 to December 2021, at the Pathology Department of Ibn Rochd University Hospital in Casablanca, Morocco, encompassing 1,426 lung cancer cases. Statistical analyses were performed using GraphPad Prism 8 software.

**Results:**

our study reveals that the mean age was 59.9±12.06 years (median: 61 years, IQR: 54-67). A clear male predominance was observed in 75.9% (n=1,083). The most common histological type was adenocarcinoma, representing 49.6% (n=708). In adenocarcinoma cases, thyroid transcription factor-1 (TTF-1) expression was positive in 95.5% (n=469). Programmed death-ligand 1 (PD-L1) was mostly negative, with a tumor proportion score (TPS) <1% in 59.7% (n=83), 1-49% in 13.7% (n=19), and ≥50% in 26.6% (n=37). Anaplastic lymphoma kinase (ALK) expression was positive in only 4.1% (n=3) of cases. Epidermal growth factor receptor (EGFR) mutations were found in 22.2% (n=24), predominantly Ex19Del in 66.6% (n=16). All ALK-positive cases were PD-L1 negative (100%, n=3), while PD-L1 expression (TPS≥1%) was detected in 38.9% (n=7) of EGFR-mutated adenocarcinomas.

**Conclusion:**

this study characterizes lung adenocarcinoma as the predominant histological type, elucidates its principal molecular features and enables the determination of possible correlations between EGFR status, PD-L1 and ALK expression among Moroccan patients.

## Introduction

Lung cancer remains a significant global health concern. In 2022, it was the most commonly diagnosed cancer worldwide, with approximately 2.5 million new cases, accounting for 12.4% of all new cancer diagnoses. It also led to the highest number of cancer-related deaths, with an estimated 1.8 million fatalities, representing 18.7% of total cancer deaths [1]. In Morocco, lung cancer is a major public health issue. According to the Global Cancer Observatory, in 2022, there were 8,825 new cases of lung cancer, making it the second most common cancer overall in the country, and it accounted for 7,970 deaths, ranking as the leading cause of cancer-related mortality [2]. Histologically, the primary forms of lung cancer comprise adenocarcinoma (ADK), small-cell carcinoma, squamous cell carcinoma and large-cell carcinoma, all of which can be classified into two general categories: small-cell lung carcinoma (SCLC) and non-small-cell lung carcinoma (NSCLC). The diagnosis of lung cancer and its histological type is based on a morphological examination, supported by the study of different markers and proteins through immunohistochemistry, such as Thyroid transcription factor-1 (TTF-1), widely used as a pulmonary ADK marker [3].

Biomarkers such as epidermal growth factor receptor (EGFR) mutations, anaplastic lymphoma kinase (ALK) rearrangements, and programmed death-ligand 1 (PD-L1) protein expression have emerged as critical tools for guiding therapeutic decision-making. Their detection through molecular profiling and/or immunohistochemical analysis allows for the stratification of patients who are likely to derive clinical benefit from targeted agents or immune checkpoint inhibitors, thereby improving clinical outcomes and personalizing therapeutic strategies [4,5]. Detailed knowledge of the epidemiological trends and molecular features of lung cancer is fundamental for improving clinical management and treatment outcomes; furthermore, it offers strategic insights to healthcare authorities, supporting evidence-based planning and more accurate forecasting of therapeutic needs and resource allocation. This study aimed to analyze the epidemiological, pathological, and molecular characteristics of lung cancer in a Moroccan cohort, with a particular focus on key biomarkers such as TTF-1, PD-L1, ALK and EGFR in lung ADK, and to investigate potential correlations among these biomarkers.

## Methods

**Study design and setting:** this retrospective observational study, conducted from January 2020 to December 2021 (24 months), aimed to investigate the demographic characteristics of patients diagnosed with lung cancer, immunohistochemical and molecular profile of lung adenocarcinoma in Moroccan patients. Data were extracted from the records of the Pathology Department at Ibn Rochd University Hospital in Casablanca, a major tertiary care and referral center that serves as one of the most prominent medical institutions in Morocco. The pathology department plays a central role in cancer diagnostics, and provides access to comprehensive and well-documented retrospective data.

**Study population:** the study population consisted of 1,426 lung cancer cases. The inclusion criteria encompassed all histologically confirmed cases of lung cancer, excluding cases with lung abnormalities without histological confirmation of malignancy and benign lung tumors.

**Data collection:** data were collected electronically by consulting patients´ medical records from the Laboratory Information System. Each case of histologically confirmed lung cancer was identified using a unique patient identification number. Information on age, sex, histological type, immunophenotype and molecular characteristics (TTF-1, PD-L1, ALK and EGFR) was extracted. The immunohistochemical analysis of TTF-1 expression was examined using the clone 8G7G3/1 and PD-L1 TPS using the clone 22C3 performed on the Dako Autostainer Link 48 platform, and ALK expression by clone D5F3 using Ventana Benchmark GX platform. Epidermal growth factor receptor mutations were detected using real-time PCR with the Cobas EGFR Mutation Test v2. A structured data extraction form was used to ensure consistency, and all collected data were entered and managed in Microsoft Excel (2019).

**Definitions:** to ensure reliable data collection and analysis, key variables were precisely defined in this study. Patient age and sex were collected as part of the demographic data, while the histological type was classified into adenocarcinoma (ADK), squamous cell carcinoma, invasive non-small cell carcinoma and neuroendocrine small cell carcinoma. Thyroid Transcription Factor-1 (TTF-1) expression was evaluated through immunohistochemistry, with a positive result defined by the presence of distinct nuclear staining in tumor cells. PD-L1 was evaluated by Tumor Proportion Score (TPS) with high expression of PD-L1 defined as TPS ≥50 %; low expression as TPS 1-49%; and negative as TPS < 1%. ALK expression was interpreted using a binary scoring system. The EGFR mutation status was determined using real-time PCR, detecting more than 60 of the most frequent variants, including the following: G719X, Ex19Del, S768I, T790M, Ex20Ins, L858R and L861Q.

**Statistical analysis:** the dataset was subjected to analysis in GraphPad Prism 8, a specialized statistical and scientific data analysis software designed for researchers. It integrates tools for data organization, statistical analysis and graphing. The variables were expressed as percentages. Epidemiological and molecular patterns were assessed through descriptive analysis of proportions. The interpretation of the results involved comparing the demographic, histological, and immunophenotypic and molecular findings with those from national and international studies, followed by an analysis to identify possible discrepancies in lung cancer profiles.

**Ethical considerations:** ethical approval for this study was obtained from the Ethics Committee of the *Centre Hospitalier Universitaire* Ibn Rochd (CHUIR) of Casablanca, under the reference number Dossier N° 3/2022 (CHUIR-EC/03-2022), dated 13 September 2022.

## Results

**General characteristics of the study population:** a total of 1,426 patients were diagnosed over the two-year period, of whom 75.9% (n=1,083) were male, yielding a sex ratio of 3.16. On average, the patients were 59.9±12.06 years old. The median age of subjects was 61 years (interquartile range: 54-67). Age distribution analysis revealed that 15.2% (n=206) of cases occurred in individuals under 50 years, 23.9% (n=324) in the 50-59 age group, 40.4% (n=548) in the 60-69 age group, and 20.5% (n=279) in those aged 70 years and above (Table 1).

**Table 1:** distribution of lung cancer cases by sex, age groups, and histological types

Characteristics	Frequency (n)	Percentage (%)
Sex (n*=1426)		
Male	1083	75.9%
Female	343	24.1%
Age group (Years) (n*=1357)		
< 50	206	15.2%
50 - 59	324	23.9%
60 - 69	548	40.4%
> 70	279	20.5%
Histology (n*=1426)		
Adenocarcinoma	708	49.6%
Squamous cell carcinoma	262	18.4%
invasive non-small cell carcinoma	170	11.9%
neuroendocrine small cell carcinoma	118	8.3%
Other	168	11.8%
n*: Number of cases with available data for the analyzed variable among the total study population		

**Histological characteristics:** the most commonly observed type was adenocarcinoma (ADK), with a prevalence of 49.6% (n=708), followed by squamous cell carcinoma with 18.4% (n=262), invasive non-small cell carcinoma with 11.9% (n=170), neuroendocrine small cell carcinoma with 8.3% (n=118), and 11.8% (n=168) for the rest of cases (Table 1).

**Figure 1:**
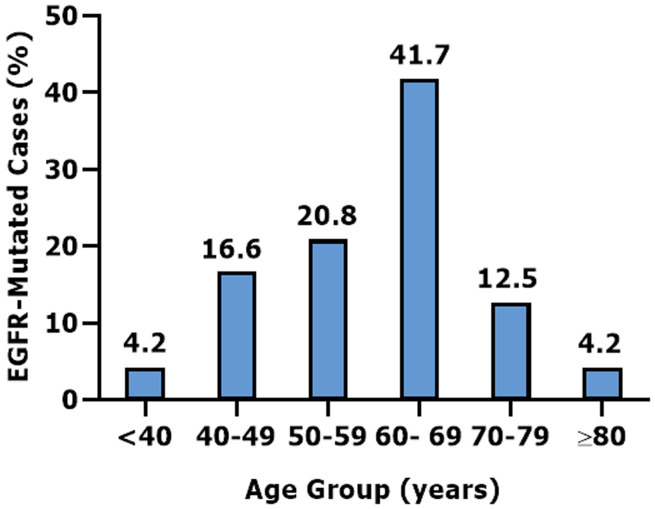
distribution of epidermal growth factor receptor-mutated adenocarcinoma cases by age groups

**Immunophenotype and molecular characteristics:** among primary ADK cases, immunohistochemical analysis revealed TTF-1 positivity in 95.5% (n=469), while 4.5% (n=22) were negative. Evaluation of PD-L1 expression showed that 59.7% (n=83) of cases had a TPS <1%, 13.7% (n=19) of cases exhibited TPS between 1% and 49%, whilst 26.6% (n=37) of cases showed a TPS ≥50%. ALK testing revealed that 95.9% (n=71) tested negative for anti-ALK antibody expression with a score of 0, and only 4.1% (n=3) tested positive (Table 2). In ALK negative cases, PD-L1 expression levels showed a TPS ≥50% in 24.2% (n=15), TPS 1% to 49% in 14.5% (n=9) and TPS <1% in 61.3% (n=38). However, all ALK-positive ADK cases (100%, n=3) were negative for PD-L1 expression (Table 3). Epidermal growth factor receptor (EGFR) analysis was conducted in 108 ADK cases; mutations were detected in 22.2% (n=24). The mutation distribution was 66.6% (n=16) for Ex19Del, 25% (n=6) for L858R and 4.2% (n=1) for the Ex20Ins. Additionally, a co-occurrence of L858R and T790M mutations was detected in 4.2% (n=1) (Table 2). Of ADK patients tested positive for EGFR mutations, patients aged <50 years (20.8%) (n=5) were less frequent compared with ≥50 years (79.2%) (n=19) (Figure 1). In EGFR-mutated ADK cases, PD-L1 expression levels showed a TPS< 1% in 61.1% (n=11), TPS 1-49% in 22.2% (n=4), and TPS ≥ 50% in 16.7% (n=3). In contrast, the PD-L1 expression in EGFR wild-type cases was distributed as follows: TPS< 1% in 57.7% (n=41), TPS 1-49% in 14.1% (n=10), and TPS ≥50% in 28.2% (n=20) (Table 3). In our study, no cases demonstrated co-occurrence of ALK expression and EGFR mutation.

**Table 2:** immunohistochemical expression of thyroid transcription factor-1, programmed death-ligand 1, and anaplastic lymphoma kinase, and epidermal growth factor receptor mutation status in lung adenocarcinoma

Biomarkers	Frequency (n)	Percentage (%)
TTF-1 expression (n*=491)		
TTF-1 Positive	469	95.5%
TTF-1 negative	22	4.5%
PD-L1 (TPS) (n*=139)		
< 1%	83	59.7%
1-49%	19	13.7%
≥ 50%	37	26.6%
ALK expression (n*=74)		
ALK positive	3	4.1%
ALK negative	71	95.9%
EGFR mutation (n*=108)		
Wild-type	84	77.8%
Mutated (Total)	24	22.2%
Exon 19 deletion	16	66.6%
Exon 21 L858R	6	25%
-With T790M	1	4.2%
Exon 20 insertion	1	4.2%
n*: number of cases with available data for the analyzed variable among the total study population; TTF-1: thyroid transcription factor-1, PD-L1: programmed death-ligand 1, TPS: tumor proportion score, ALK: anaplastic lymphoma kinase, EGFR: epidermal growth factor receptor		

**Table 3:** distribution of programmed death-ligand 1 expression according to anaplastic lymphoma kinase status and epidermal growth factor receptor mutation status in lung adenocarcinoma

Category	PD-L1 expression status (n (%))		
	TPS <1%	TPS 1-49%	TPS ≥50%
ALK expression (n*=65)			
ALK positive (n=3)	3 (100%)	0 (0%)	0 (0%)
ALK negative (n=62)	38 (61.3%)	9 (14.5%)	15 (24.2%)
EGFR expression (n*=89)			
Wild-type EGFR (n=71)	41 (57.7%)	10 (14.1%)	20 (28.2%)
Mutated EGFR (n=18)	11 (61.1%)	4 (22.2%)	3 (16.7%)
n*: number of cases with available data for the analyzed variable among the total study population. PD-L1: programmed death-ligand 1, TPS: tumor proportion score, ALK: anaplastic lymphoma kinase, EGFR: epidermal growth factor receptor.			

## Discussion

Our study aimed to analyze the epidemiological and molecular characteristics of lung cancer in Moroccan patients, with a specific focus on the histological type, immunophenotypic profile, and molecular features of pulmonary adenocarcinoma (ADK). Findings indicated that ADK was the most common type 49.6%, with TTF-1 positivity in 95.5%, PD-L1 TPS <1% in 59.7%, and ALK negativity in 95.9%. EGFR mutations were identified in 22.2% of ADK cases, with Ex19Del being the most frequent variant. Of the 1,426 cases analyzed in our study, 75.9% were predominantly male. Possible explanations for this could include smoking habits, early onset of smoking, and men's high-risk occupations. This predominance is similar to that observed in the Greater Casablanca Cancer Registry in 2008-2012 [6]. The 60-69 age group was the most affected (40.4%). Previous studies have reported similar age distributions, 48.1% in Morocco within the 60-69 age group [7], and China with the highest proportion of cases observed in the 65-69 age group [8]. This trend aligns with the increased cancer risk associated with aging, likely due to accumulated DNA damage from environmental and biological factors [9].

The incidence of the various histological types of lung cancer has changed in recent years, with ADK now being the most prevalent type in both smokers and non-smokers, as well as in males and females. Consistent with international patterns, ADK was the predominant histological subtype in our study with 49.6%, followed by squamous cell carcinoma with 18.4%, aligning with previous research across Jordan, China, and India, demonstrating the prevalence of ADK with respective percentages of 55.3%, 34.3% and 56.4%. Within these studies, squamous cell carcinoma was identified as the second most frequent histological type, accounting for 30.2%, 14.5% and 25.6% respectively, and small cell lung cancer is ranked third [8,10,11]. Immunohistochemical analysis was performed on ADK cases, as it allows for the evaluation of prognostic and predictive markers relevant to treatment response [12]. Thyroid transcription factor-1 expression was observed in 95.5 % of ADK cases. This positivity rate in our study was consistent with findings from studies conducted in Korea at 80.3% and Japan at 65.5% [13,14], reinforcing its role as a reliable diagnostic marker for pulmonary ADK across diverse populations. In addition of its diagnostic role as a routine identifier of lung ADK, TTF-1 may also serve as a predictive or prognostic marker for patients with this type of cancer [15].

The PD-L1 expression rate in our study (40.3%) aligns with the literature [16], and reflects its clinical relevance, particularly given that PD-L1 remains the only FDA-approved biomarker for guiding immune checkpoint inhibitor therapy in ADK. Its established role in first- and second-line treatment of advanced-stage NSCLC underscores the importance of routine assessment in clinical practice [17,18]. Anaplastic lymphoma kinase expression was negative in 95.9% of primary ADK cases tested in our study, scoring a 0. In contrast, it was positive in 4.1% of cases. Nonetheless, the incidence of ALK rearrangements in NSCLC patients is about 5-6% according to literature; their presence is associated with younger age, never smoking and the presence of metastases in the central nervous system [19,20]. It is therefore becoming increasingly important to identify driver alterations in order to guide treatment with targeted therapies. In our study, all ALK-positive ADK cases demonstrated negative PD-L1 expression (TPS <1%), while 61.3% of ALK-negative cases also exhibited similarly negative expression. These findings are consistent with previous studies, one of which reported no significant difference in PD-L1 expression between ALK wild-type and translocated cases [21], and another that found no significant association between ALK status and PD-L1 expression (TPS ≥1%) [22].

Targetable driver mutations in NSCLC have significantly improved treatment outcomes and prognosis [23], including EGFR mutations observed in 22.2% of ADK cases in our study (66.6% for the Ex19Del, 25% for L858R mutation), consistent with a Moroccan study reporting 21.9%, mainly in exon 19 deletion and 21 L858R (65.8% and 17.8%, respectively) [24]. Epidermal growth factor receptor mutation rates across North Africa show some variation [25-27], likely due to population and methodological differences. The EGFR mutation rate observed in our study reflects Morocco´s intermediate position, with frequencies between higher rates in Southern Asia (46%) and lower rates in South America, North America, and Europe (7.9%, 9.2%, 13.4%, respectively) [28]. This intermediate EGFR mutation prevalence observed in North Africa may be attributed to its mixed genetic background, resulting from historical migrations. The correlation between age and oncogenic alterations has been explored across cancers. In our study, we investigated the potential association between EGFR mutations and patient age in lung ADK. The majority (79.2%) of EGFR-mutated cases were observed in patients aged ≥50 years, which was consistent with previous studies [29,30]. This finding may be indicative of age-related heterogeneity in tumor biology, consistent with the known impact of aging on cancer risk [31].

In our study, PD-L1 expression rates were slightly lower in EGFR-mutated ADK (38.9%) compared to EGFR wild-type (42.3%). This aligns with a meta-analysis reporting a negative correlation between EGFR mutations and PD-L1 expression in NSCLC [32]. Similarly, a recent study from Turkey found higher PD-L1 TPS in EGFR wild-type cases [33]. These findings suggest a possible inverse relationship; however, the association remains complex and may be influenced by additional factors such as the tumor microenvironment and the specific EGFR mutation subtype. The limitations of this study were the lack of specific information from the patient files, such as exposure to environmental risk factors, which would have enabled an analysis of their relationship with the histological type. The strengths of this study include its significant sample size, the detailed characterization of lung cancer cases, and the comprehensive analysis of both epidemiological and molecular features. The integration of biomarker expression data contributes to a better understanding of the molecular profile of lung adenocarcinoma in the Moroccan population, offering valuable insight for future research and clinical decision-making.

## Conclusion

This study delineates the epidemiological and molecular characteristics of lung cancer among Moroccan patients, highlighting lung ADK as the predominant histological type. It elucidates immunophenotype and key molecular characteristics, including TTF-1 expression, PD-L1 expression and ALK rearrangements, along with their respective frequencies and shows that the level of EGFR mutation is between Asia and Europe/America, with a negative correlation identified between EGFR mutations and PD-L1 expression, and no significant difference in PD-L1 expression between ALK wild-type and ALK-rearranged ADK cases. A comprehensive knowledge of histopathological subtypes and epidemiological factors is crucial to personalize therapeutic approaches, enhance clinical decision-making, and refine prognostic evaluations. Aligning with the literature, these findings provide valuable insights to refine lung cancer management, support targeted therapy development, and guide evidence-based prevention strategies in Morocco.

### What is known about this topic

- Lung cancer is the leading cause of cancer-related mortality worldwide and in Morocco, accounting for the highest mortality rates among both men and women;
- In Morocco, it is the most prevalent cancer among men;
- Adenocarcinoma is the most common type of lung cancer.

### What this study adds

- This study offers a general overview of the epidemiology of lung cancer in Morocco;
- Our findings present detailed insights into the molecular characteristics of lung adenocarcinoma among Moroccan patients;
- Analyze the correlations between EGFR status, PD-L1 and ALK expression of lung adenocarcinoma in Morocco.
